# Non-Fluoroscopic Radiofrequency Ablation of Left Atrial Appendage Tachycardia During Early Pregnancy

**DOI:** 10.21470/1678-9741-2018-0370

**Published:** 2019

**Authors:** Wenxiu Liu, Shuying Qi, Heng Cai, Dongmei Wang, Leisheng Ru

**Affiliations:** 1 Department of Cardiology, Bethune International Peace Hospital, Shijiazhuang, Hebei, China.; 2 Department of Cardiology, General Hospital of TianJin Medical University, TianJin, China.

**Keywords:** Ectopic Atrial Tachycardia, Cardiovascular Pregnancy Complications, Catheter Ablation, Cardiac Arrhythmias - Therapy, Atrium, Cardiac Electrophysiology - Methods

## Abstract

Management of symptomatic atrial tachycardia (AT) during pregnancy seems challenging, especially those originating from left atrial appendage (LAA), which easily tend to be incessant and mediate cardiomyopathy. It’s contradictory between therapy and pregnancy. In this study, we report a case of a woman who presented with persistent AT, which lead to heart failure, during early pregnancy. She underwent successful catheter ablation using CartoSound and electroanatomic mapping without fluoroscopy. An electrophysiology (EP) study confirmed a focal LAA tachycardia. Soon after, left ventricular function of her heart normalized, and the patient successfully delivered a healthy child.

**Table:** 

Abbreviations, acronyms & symbols	
AT	= Atrial tachycardia
ACC	= American College
AHA	= Cardiology and American Heart Association
CS	= Coronary sinus
ECG	= Electrocardiogram
EP	= Electrophysiology
FAM	= Fast anatomical mapping
LAA	= Left atrial appendage
LVEDD	= Left ventricular end-diastolic dimension
LVEF	= Left ventricular ejection fraction
RA	= Right atrium
RF	= Radiofrequency
SVT	= Supraventricular tachycardia

## INTRODUCTION

The left atrial appendage (LAA) is a rare site of origin for ectopic atrial tachycardia (AT). It often presents as incessant tachycardia, which easily mediates cardiomyopathy. And it’s always refractory to drugs. Meanwhile, tachycardia from LAA presents a unique challenge for successful ablation because of special anatomical location. In this report, we describe an ablation for AT originating from LAA curing an early pregnant woman without fluoroscopy.

## CASE REPORT

**Fig. 1:**
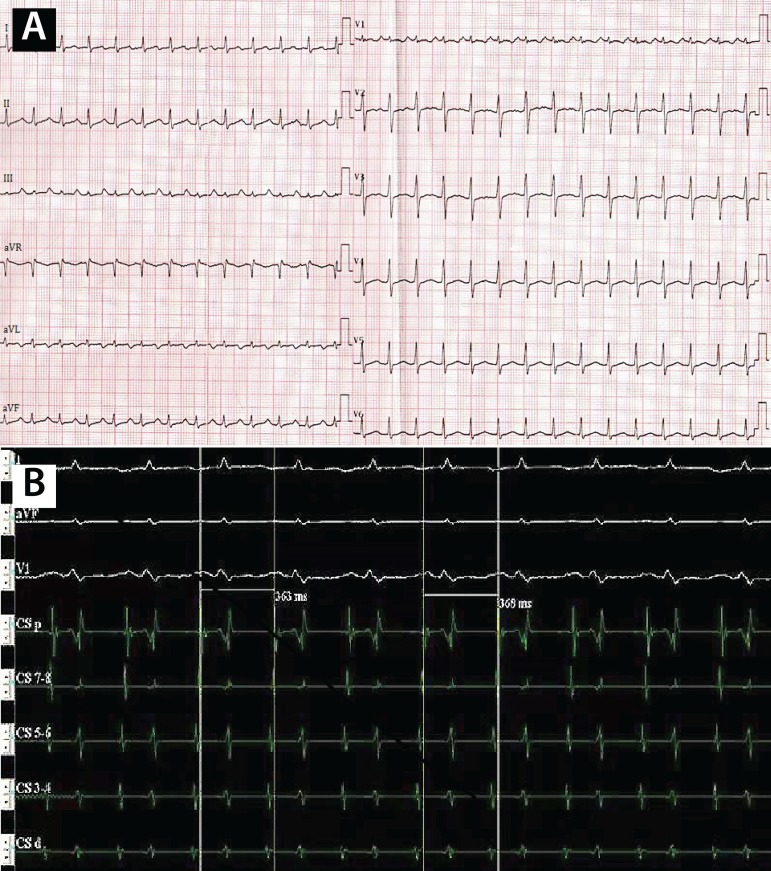
A) Surface ECG shows narrow QRS and long RP tachycardia, with frequency of 150 bpm. It notes the upright P-wave in leads II, III and aVF, inverted P-wave in leads I and aVL, and an upright P-wave in lead V1. B) Intracardiac ECG shows long RP tachycardia, with A-A interval of 363-368 ms. The activation sequence of mapping electrode in CS reveals the earliest atrial activation of CSd.

A 29-year-old woman was admitted with a 2-week history of progressive paroxysmal palpitations accompanied by persistent chest tightness and dyspnea. She had no other medical history. During her 3 years of marriage, she had encountered difficulty achieving pregnancy naturally. When admitted, she was in the 11^th^ week of gestation following in vitro fertilization. Physical examination revealed a pulse of 167 beats per minute (bpm), regular heart rhythm, clear heart sounds with no pathological murmur and no lower limb edema. Auxiliary examinations included a 12-lead electrocardiogram (ECG) which showed AT at 150 bpm with a negative *P*-wave in leads I, aVL and aVR, as well as a positive *P*-wave in leads II, III, aVF and V_1_ (Figure1A), and an echocardiogram which revealed an LA of 42mm (anteroposterior diameter), left ventricular end-diastolic dimension (LVEDD) of 55mm and left ventricular ejection fraction (LVEF) of 48%. She was found to be euthyroid and her N-terminal pro-brain natriuretic peptide level was 4285 pg/ml. The patient was diagnosed with atrial tachyarrhythmia. An esophageal lead was applied to confirm the diagnosis, but rapid pacing failed to terminate it. Rest ECG showed a rate of 110 to 120 bpm. Slight movement, such as eating or going to the toilet, caused her heart rate to exceed 150 bpm. Intravenous administration of propafenone transiently converted the tachycardia to sinus rhythm but could not sustain. Other medications such as metoprolol could not adequately control the heart rate either.

**Fig. 2:**
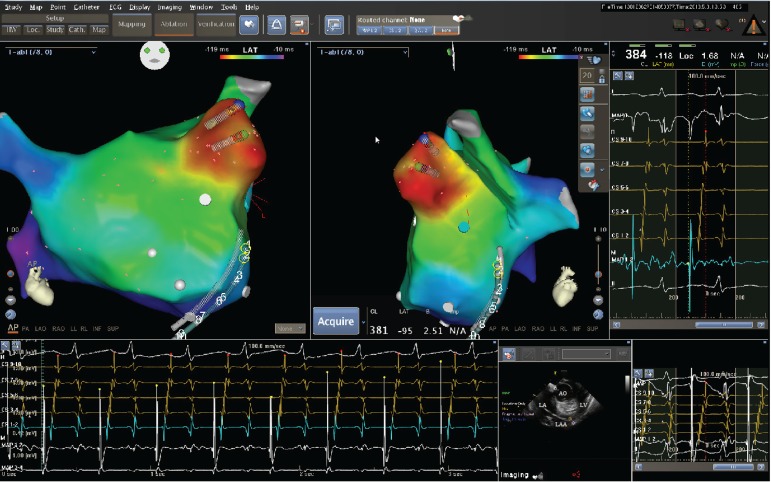
The activation map shows that left atrial appendage is the origin of tachycardia, namely the focal atrial tachycardia originating from left atrial appendage. Location of CartoSound ablation catheter--the catheter is closely against the tissue. LAA=left atrial appendage; LA=left atrium; AO=aorta; LV=left ventricle

Ablation was performed under local anesthesia, Multichannel EP recorder (EP MedSystems, USA) and three-dimensional (3D) electroanatomic mapping system (CartoSound, Biosense Webster, CA, USA). The patient still exhibited tachycardia while undergoing the procedure. The right femoral vein was cannulated using an 8French (F) sheath, through which a saline infusion ablation catheter (ThermoCool Electrophysiology Catheter, Biosense Webster, CA, USA) was introduced into the right atrium (RA). A detailed electro-anatomic map of the RA and coronary sinus (CS) were created using fast anatomical mapping (FAM). Then the right internal jugular vein was cannulated using a 6F sheath through which a decapolar EP catheter (Rithm ID, Synaptic Medical, CN) was introduced into the CS under the guidance of the electro-anatomic map. The intracardiac electrogram showed a cycle length of 330-370 msec and CS activation order of CS_1,2_ earlier than CS_9,10_ (Figure1B), which confirmed the AT originating from the LA. Then the right femoral vein was cannulated using a 10F sheath and an 8.5F long sheath, through which an EP catheter (SoundStar, Biosense Webster, CA, USA) was introduced and a transseptal needle (St. Jude Medical, USA) was applied to perform transseptal puncture under CartoSound guidance. Four thousand units of unfractionated heparin were administered intravenously with access to LA to maintain an active clotting time of approximately 350 seconds. The ablation catheter and a circular decapolar catheter (Lasso, Biosense Webster, CA, USA) were introduced into the LA via the transseptal site. A detailed electro-anatomic map of LA was created and clearly demonstrated focal activation originating from the very distal LAA (Figure2). There was no evidence of macro-reentry. Radiofrequency (RF) ablation was performed using 20 watts, titrating up to 25 watts, for approximately 16 times (power control mode, 40℃, 30cc/min). Each time, ablation energy was delivered for 6-10 seconds. Ablation at this site terminated the tachycardia with no recurrence noted during a 30-minute observation period. No tachycardia was induced by programmed stimulation and rapid atrial pacing. The procedure lasted for 2.5 hours with no fluoroscopy and without complications.

At the 1-month follow-up visit, the ECG showed normal sinus rhythm and the echocardiogram showed LA of 30 mm, LV of 48 mm, LVEF of 52%. Three months after the procedure, a 24-hour Holter exam revealed sinus rhythm with 7987 atrial premature beats and 903 episodes of paroxysmal AT (8.1% of the day). And the patient complained of occasional palpitations. Six months later, a 24-hour Holter exam conducted showed sinus rhythm with 37 atrial premature beats and 5 episodes of paroxysmal AT (0.06% of the day). A healthy baby boy was eventually delivered by Cesarean section. No palpitations were reported thereafter.

## DISCUSSION

AT is a relatively rare supraventricular tachycardia (SVT) with a reported prevalence of 5-10%. Focal atrial tachycardia can arise from various portions of the atrium. These include the crista terminalis, around or inside the CS, near or inside the pulmonary veins, superior vena cava, atrial septum, and Koch’s triangle. LAA tachycardia is uncommon, accounting for only 3% of all focal AT. And AT originating from LAA is likely to become persistent^[1]^. This patient’s initial presentation was due to cardiomyopathy secondary to persistent tachycardia. Meanwhile, it has been reported that the recurrence rate of this kind of AT is as high as 30%^[2]^. No data exists regarding incidence, ablation therapy or prognosis of AT originating from the LAA in pregnant women. Review of the literature showed that in 2015^[3]^, among 27 cases of ablation therapy performed for arrhythmias during pregnancy, two cases of AT originating from the right atrial appendage were treated operatively under x-ray guidance. No cases of AT originating from the LAA were described.

As persistent tachycardia reduces mother’s cardiac output, the resulting decrease in uteroplacental perfusion raises the likelihood of fetal distress and developmental retardation. Physical therapy, such as esophageal pacing and electrical cardioversion, is simple and repeatable, but has limited efficiency and is associated with high rates of recurrence. All antiarrhythmic drugs pose risks of fetal adverse effects or teratogenicity. Therefore, definitive therapy for these patients, either with catheter ablation or drug therapy, is usually deferred until the postpartum period. In fact, the American College of Cardiology and American Heart Association (ACC/AHA) guidelines recommend that ablation be considered in pregnant women with drug-refractory, poorly-tolerated SVTs. RF catheter ablation is effective, but the associated radiation also has teratogenic effects^[4,5]^. In recent years, with the use of 3D-mapping systems, RF catheter ablation without fluoroscopy has brought new hope to special patients, such as pregnant women and children.

Electroanatomic navigation systems can comfortably depict the activation of all chambers mapped and allow reproducible ablation catheter repositioning at the “spot of interest”. In this case report, we have described ablation under CartoSound system. The CartoSound system provided a considerably safer and more efficient technique compared with conventional methods. The procedure included a double transseptal puncture, activation mapping and successful ablation of a LAA focal tachycardia. All of these were performed without any fluoroscopy. To the best of our knowledge, this report is the first to describe LAA ablation performed in a pregnant patient without fluoroscopy.

We conclude that catheter ablation may be safely and successfully performed using the CartoSound 3D-mapping system guidance without radiation in special groups. Catheter ablation therefore should be considered for these patients with arrhythmias refractory to medical therapy.

**Table:** 

Author's roles & responsibilities	
WL	Participated in the operation, drafted the work and revise it; final approval of the version to be published
SQ	Participated the operation, and agree to be accountable for all aspects of the work; final approval of the version to be published
HC	Participated the operation; final approval of the version to be published
DW	Participated in revising the work; final approval of the version to be published
LR	Provided final approval of the version to be published; final approval of the version to be published
